# From Chaos to Ordering: New Studies in the Shannon Entropy of 2D Patterns

**DOI:** 10.3390/e24060802

**Published:** 2022-06-08

**Authors:** Irina Legchenkova, Mark Frenkel, Nir Shvalb, Shraga Shoval, Oleg V. Gendelman, Edward Bormashenko

**Affiliations:** 1 Department of Chemical Engineering, Engineering Faculty, Ariel University, P.O. Box 3, Ariel 407000, Israel; ilegchenkova@gmail.com (I.L.); markfr@ariel.ac.il (M.F.); 2 Department of Mechanical Engineering & Mechatronics, Faculty of Engineering, Ariel University, P.O. Box 3, Ariel 407000, Israel; nirnirnir121212@gmail.com; 3 Department of Industrial Engineering and Management, Faculty of Engineering, Ariel University, P.O. Box 3, Ariel 407000, Israel; shraga@ariel.ac.il; 4 Faculty of Mechanical Engineering, Technion—Israel Institute of Technology, P.O. Box 10, Haifa 3200003, Israel; ovgend@tx.technion.ac.il

**Keywords:** voronoi tessellation, shannon entropy, random set of points, ordering, lamellae, spherulite, continuous measure of symmetry

## Abstract

Properties of the Voronoi tessellations arising from random 2D distribution points are reported. We applied an iterative procedure to the Voronoi diagrams generated by a set of points randomly placed on the plane. The procedure implied dividing the edges of Voronoi cells into equal or random parts. The dividing points were then used to construct the following Voronoi diagram. Repeating this procedure led to a surprising effect of the positional ordering of Voronoi cells, reminiscent of the formation of lamellae and spherulites in linear semi-crystalline polymers and metallic glasses. Thus, we can conclude that by applying even a simple set of rules to a random set of seeds, we can introduce order into an initially disordered system. At the same time, the Shannon (Voronoi) entropy showed a tendency to attain values that are typical for completely random patterns; thus, the Shannon (Voronoi) entropy does not distinguish the short-range ordering. The Shannon entropy and the continuous measure of symmetry of the patterns demonstrated the distinct asymptotic behavior, while approaching the close saturation values with the increase in the number of iteration steps. The Shannon entropy grew with the number of iterations, whereas the continuous measure of symmetry of the same patterns demonstrated the opposite asymptotic behavior. The Shannon (Voronoi) entropy is not an unambiguous measure of order in the 2D patterns. The more symmetrical patterns may demonstrate the higher values of the Shannon entropy.

## 1. Introduction

The quantification of ordering appearing in 2D sets of objects is important from both fundamental and engineering points of view. The quantification of 2D ordering is crucial for understanding phase transitions [1,2,3,4], the characterization of attractors in non-linear systems [5], the treatment of images [6] and machine learning applied for study of physical systems [7,8,9]. Various measures and mathematical procedures were implemented for the quantification of ordering in 2D patterns, including Voronoi tessellations followed by the calculation of the information (Shannon) entropy of the distribution of the Voronoi polygons (which is also called the Voronoi entropy and abbreviated in the text as VE; VE below within the text denotes the Shannon entropy) [10,11,12,13,14,15,16,17], Minkovski functionals [18,19,20], method of correlation functions [21,22,23], and calculation of the recently introduced continuous and Shannon measures of symmetry [6,24,25,26,27]. It was demonstrated that the introduced measures of symmetry do not necessarily correlate [27,28]. Moreover, they may demonstrate anti-correlation. For example, the maxima of the continuous measure of symmetry (abbreviated CSM), calculated for the levitating droplet clusters, may correspond to the minima of the VE of the same cluster [28]. The analysis of the Penrose tiling provides an even more convincing example. It was demonstrated that the continuous symmetry measure and the Shannon (Voronoi) entropy of the studied sets of points, generated by the Penrose tiling, do not correlate [29]. We, therefore, conclude that the notion of “ordering”, as applied to the characterization of 2D patents built of points, has a fine structure, and cannot be quantified by a single numerical parameter. In the presented paper, we study the fascinating phenomenon of ordering that emerges from an initial random 2D set of points and compare the Shannon (Voronoi) entropy and the continuous measure of symmetry of the addressed patterns.

## 2. Methods

### 2.1. Software

We used MATLAB software to generate sets of randomly located seeds, build and evaluate properties of the corresponding Voronoi tessellations, and divide the edges of the Voronoi cells. We also used the open-access software designed at the Department of Physics and Astronomy at the University of California, Irvine, which is available on the website https://www.physics.uci.edu/~foams/do_all.html (accessed on 1 March 2022). The software enabled exact identification of the polygons constituting the given Voronoi tessellation.

### 2.2. Procedure for Generation of Voronoi Diagrams

**Figure 1:**
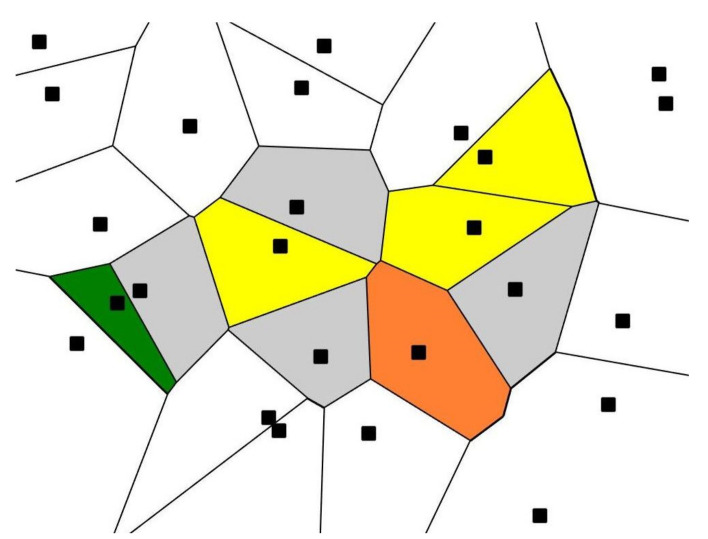
Voronoi diagram (tessellation) emerging from the initial 25 randomly placed points (black squares); green polygon is a quadrangle; yellow polygons are pentagons; grey polygons are hexagons; the orange polygon is an octagon.

We started with a set of 25 points randomly located on the plane (seeds), as shown in Figure 1. Next, the Voronoi tessellation generated by the random seeds was built. A Voronoi tessellation of an infinite plane is a partitioning of the plane into regions based on the distance to a specified discrete set of points (called seeds or nuclei and shown with black squares in Figure 1). For each seed, there is a corresponding region, consisting of all points closer to that seed than to any other [10,11,12,13,14,15,16,17].

Next, the following iterative procedure was applied to the initial random Voronoi tessellation depicted in Figure 1 according to the following steps: the edges forming the Voronoi polygons were divided into two or three parts, equally or randomly. The dividing points were then taken as the new seeds for new Voronoi tessellations (the former seeds were excluded). This new diagram, in turn, was subjected to the division of the edges of the polygons. The experiment was carried out for 10 different initial sets of random points. The boundary open shapes recognized in Figure 1 were excluded under the quantitative treatment of the Voronoi tessellations.

A random number generator of the MATLAB software was used for the generation of the initial sets of coordinates of uniformly distributed random seeds in the interval (0, 1). Ten sets of the coordinates resulting in the initial Shannon (Voronoi) entropy of the set restricted within $0.950<S_{vor}<1.735$ were selected as initial sets for the aforementioned iterative procedure.

### 2.3. Procedure of Calculation of the Continuous Measure of Symmetry

**Figure 2:**
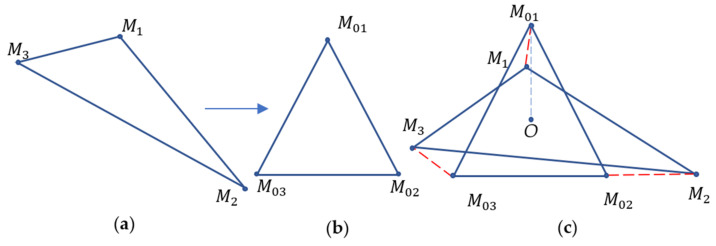
Given non-symmetric triangle $\;M_{1}M_{2}M_{3}$ (**a**). The equilateral triangle $\;M_{01}M_{02}M_{03}$ represents the symmetrical shape corresponding to the non-symmetric triangle $\;M_{1}M_{2}M_{3}$. (**b**) The equilateral triangle $M_{01}M_{02}M_{03}$ is depicted. Calculation of the CSM where point *O* is the common centroid is shown (**c**).

Now we introduce the continuous symmetry measure (abbreviated CSM), as it was defined in references [24,25,26]. Consider a non-symmetrical shape consisting of $n_{k}$ points $M_{i}$, $\left(i=1,2\ldots n_{k}\right)$ (which are the seed points of the given Voronoi diagram) characterized by a given symmetry group *G*. The continuous symmetry measure denoted $S\left(G\right)$ is determined by the minimal average square displacement of the points $M_{i}$ that the shape must undergo in order to acquire the prescribed *G*-symmetry. Assume that the *G*-symmetrical shape, emerges from the set of points ${\hat{M}}_{i}$. Since the set ${\hat{M}}_{i}$ is established, a CSM is defined as

(1) $$S\left(G\right)=\frac{1}{n_{p}}\sum_{i=1}^{n_{p}}{\left|M_{i}-{\hat{M}}_{i}\right|}^{2}$$

(the square values in Equation (2) provide a function that is isotropic, continuous, and differentiable). At the first step, the points of the nearest shape possessing the *G*-symmetry must be identified. An algorithm that identifies the set of points ${\hat{M}}_{i}$ that constitutes this symmetrical shape was introduced in references [24,25,26]. Figure 2 depicts an equilateral triangle $\;M_{01}M_{02}M_{03}$ representing the symmetric shape that corresponds to the given non-symmetric triangle $\;M_{1}M_{2}M_{3}$.

The details of the transformation of the non-symmetric triangle $\;M_{1}M_{2}M_{3}$ to the symmetric equilateral triangle $\;M_{01}M_{02}M_{03}$ and algorithm of the calculation of the continuous measure of symmetry are supplied in references [27,28,29].

## 3. Results

### 3.1. Ordering in Voronoi Diagrams: Qualitative Analysis

**Figure 3:**
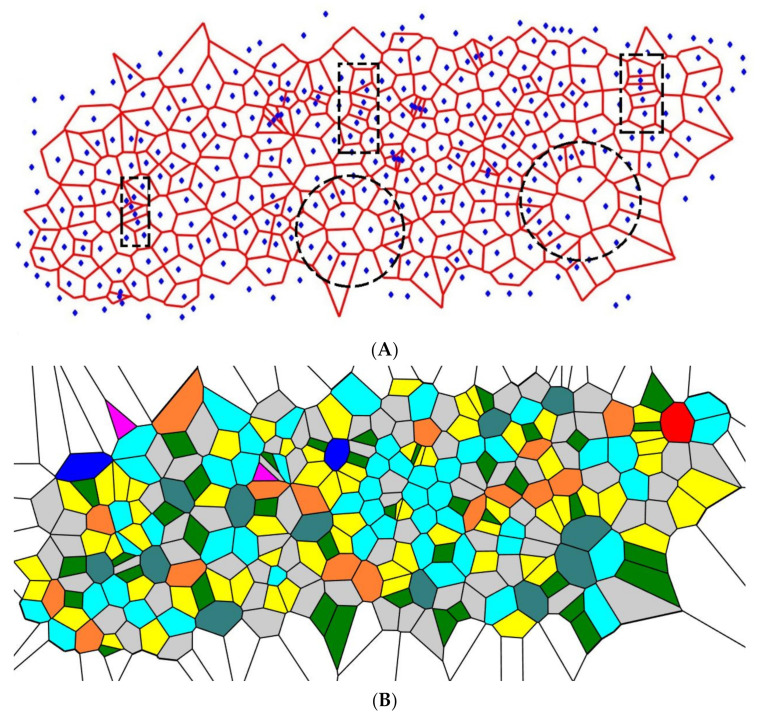
The pattern emerging from the initial random Voronoi tessellation is depicted. Centers of the initial Voronoi tessellation edges were taken as the seeds for a new one; the former seeds were excluded. The pattern emerging from the four-time repetition of the procedure is depicted. (**A**) Short-range ordering appearing in the pattern is illustrated with the dashed figures: lamellae-like structures are circled by black dashed rectangles; spherulite is shown with the dashed circle. (**B**) Polygons inherent for the Voronoi four-time tessellation are depicted: purple polygons are triangles; green polygons are quadrangles; yellow polygons are pentagons; grey polygons are hexagons; blue (aqua) polygons are heptagons; orange polygons are octagons; teal polygons are nonagons; red polygons are decagons; deep blue polygons have 11 and more vertices.

Consider the division of the Voronoi cells’ edges into two equal parts. The procedure of generating new seeds was repeated four times, giving rise to the patterns depicted in Figure 3. The peculiarities inherent to this pattern were typical for all of the initial sets used. The patterns depicted in Figure 3 somewhat surprisingly demonstrate the short-range order (recall that the initial set of points was random). Two types of order appear in the patterns depicted in Figure 3, which we call lamellae-like structures (circled with dashed black rectangles in Figure 3) and spherulite-like patterns (circled with dashed black circles in Figure 3), built on the aforementioned lamellae. This type of self-organization is inherent in the melt-crystallized polymers, such as polypropylene [30,31]. Suppose a molten linear polymer (such as polypropylene or polyethylene) is cooled down slowly. In that case, some polymer chains take on a certain orderly configuration: they align themselves in semicrystalline plates called lamellae [30,31].

Spherulites are acicular structures radiating from a common core. Spherulites appear not only in polymer science but also in geology [32,33,34] and biology (calcium carbonate spherulites) as a result of animal digestive processes [35]. In geology, spherulitic aggregate are radiating arrays of fibrous, needle-like or acicular crystals that are common in glassy felsic volcanic rocks [32,33,34]. In polymer science, spherulites are defined as spherical semicrystalline regions inside non-branched linear polymers [30,31,36]. Their formation is associated with the crystallization of polymers from the melt and is controlled by several parameters, such as the number of nucleation sites, structure of the polymer molecules and cooling rate [30,31,36]. Depending on those parameters, the spherulite diameter may vary in a wide range from a few micrometers to millimeters. Spherulites are composed of highly ordered lamellae (thin ordered plates), which result in higher density, hardness, but also brittleness when compared to disordered regions in a polymer.

Spherulite-like self-organization is clearly recognized in the pattern, shown in Figure 3. The lamellae in non-branched polymers are connected by amorphous (disordered) regions; this type of a structure is also inherent in the reported Voronoi tessellations. It should be emphasized that spherulites appear in Voronoi tessellations built according to the aforementioned iterative procedure, regardless of the initial random distribution of the seed points. The physicochemical mechanisms of formation and ordering in polymer spherulites are still unknown and controversial [36]. The mechanism of self-organization in the studied patterns in its relation to the formation of spherulites in non-branched polymers is discussed below in detail.

So we recognized that the procedure mentioned above for the construction of the Voronoi tessellations gave rise to the distinct polymer-like short range ordering in the Voronoi diagrams arising from the initial random 2D distribution of points. What is the reason for this surprising ordering? We hypothesized first that the effect of ordering emerged from the equal division of the edges of the Voronoi cells. In order to check this hypothesis, we carried out an iterative procedure under which the edges of the Voronoi polygons were divided into two *random* parts.

**Figure 4:**
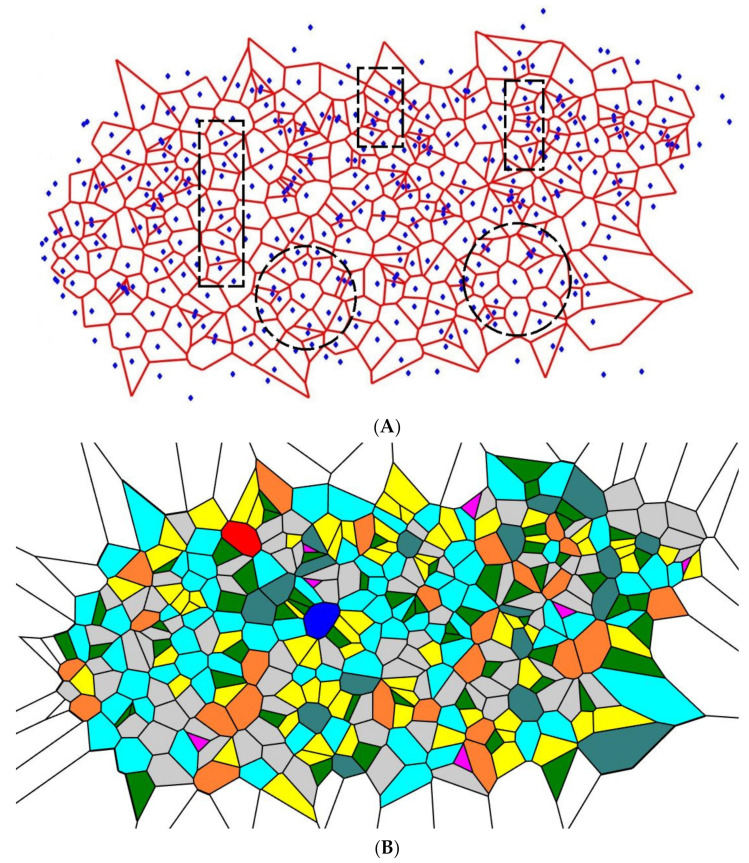
The pattern emerging from the initial random Voronoi tessellation after four-time repetitions of the procedure of random division of the polygons’ sides is depicted (**A**) Short-range ordering appearing in the pattern is illustrated with the dashed figures: lamellae-like structures are circled by black dashed rectangles; spherulites are shown with the dashed circles. (**B**) Polygons inherent for the Voronoi tessellation are depicted: purple polygons are triangles; green polygons are quadrangles; yellow polygons are pentagons; grey polygons are hexagons; blue (aqua) polygons are heptagons; orange polygons are octagons; teal polygons are nonagons; red polygons are decagons; deep blue polygons have 11 and more vertices.

After four-time repetition of the procedure, the random division of the edges of the Voronoi tessellations yielded the patterns depicted in Figure 4. Again, the similar short-range order is distinguished in the pattern, the same types of the short-range order appear in the patterns shown in Figure 4, namely, the lamellae-like structures (circled with dashed black rectangles) and spherulite-like patterns built of lamellae (circled with dashed black circles in Figure 4). Again, spherulites and lamellae appeared in the Voronoi tessellations, regardless of the initial random distribution of the seed points. Thus, we conclude that the random partition of the edges appearing in the Voronoi tessellation emerging from the random initial distribution of points also give rise to the crystalline-polymer-like patterns, demonstrating the short-range order.

**Figure 5:**
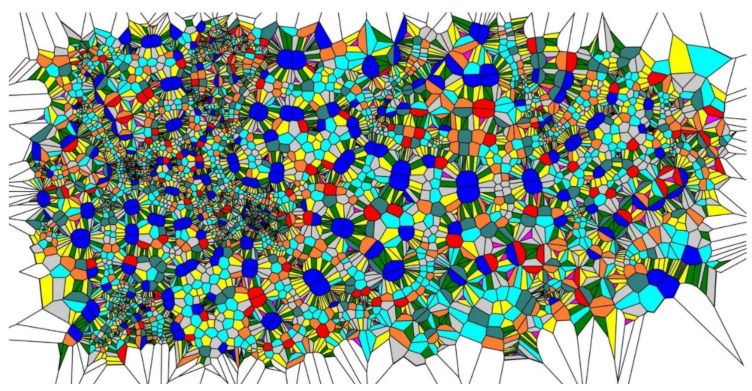
The pattern emerging from the initial random Voronoi tessellation is depicted. Edges of the initial Voronoi tessellation were divided into three equal segments; the dividing points were taken as the seeds for a new tessellation; the former seeds were excluded. The pattern emerging from the four-time repetition of the procedure is depicted. Spherulites and lamellae are distinctly distinguished within the pattern. Marking of the polygons is as follows: purple polygons are triangles; green polygons are quadrangles; yellow polygons are pentagons; grey polygons are hexagons; blue (aqua) polygons are heptagons; orange polygons are octagons; teal polygons are nonagons; red polygons are decagons; deep blue polygons have eleven and more vertices.

We continued our investigations and performed the same procedure, which differed from the aforementioned ones, in the division of the edges of the Voronoi tessellation arising from the initial random distribution of points into three equal sections. Again, spherulites and lamellae are distinctly recognized from the pattern, shown in Figure 5. The pattern depicted in Figure 5 is the most interesting result of our simulations. Again, the pattern demonstrates amazing similarity to the structures typical for crystalline polymers ordered under slow cooling of the polymer melt.

**Figure 6:**
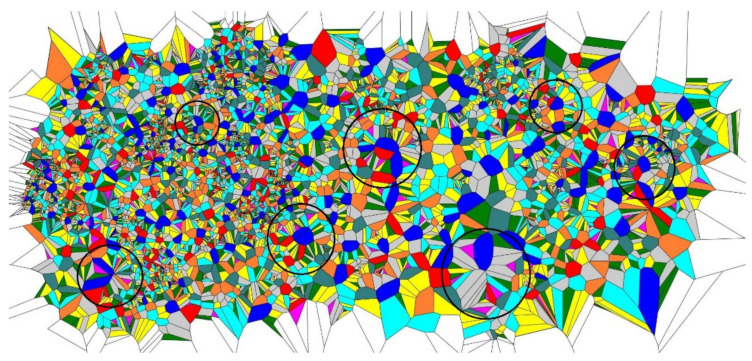
The pattern emerging from the initial random Voronoi tessellation is depicted. Three points randomly placed on the edges of the initial Voronoi tessellation were taken as the seeds for a new tessellation; the former seeds were excluded. The pattern emerging from the four-time repetition of the procedure is depicted. Spherulites are circled. Polygons inherent for the Voronoi tessellation are depicted: purple polygons are triangles; green polygons are quadrangles; yellow polygons are pentagons; grey polygons are hexagons; blue (aqua) polygons are heptagons; orange polygons are octagons; teal polygons are nonagons; red polygons are decagons; deep blue polygons have 11 and more vertices.

In the next experiment, the same set of randomly placed points was also subjected to the iterative procedure with division of the polygons’ edges into three random parts. The process was repeated four times. This procedure yielded the eventual pattern, shown in Figure 6. In this case, also spherulite-like structures (circled in Figure 6) emerged as well; however, the ordering in this case is less pronounced, when compared with the pattern depicted in Figure 5.

### 3.2. Quantitative Analysis of the Voronoi Diagrams

**Figure 7:**
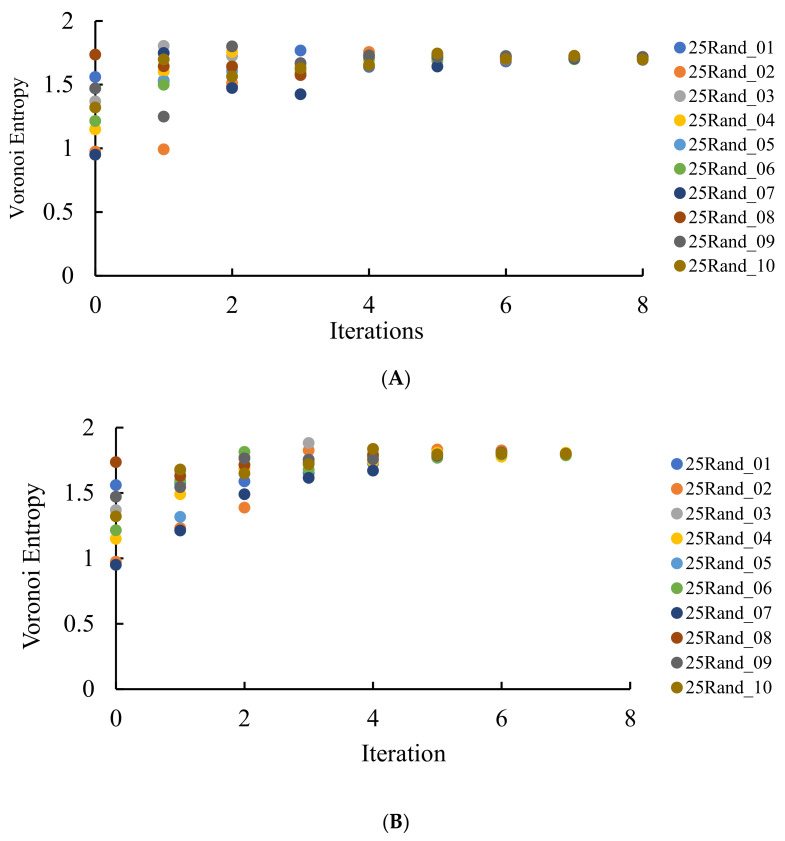

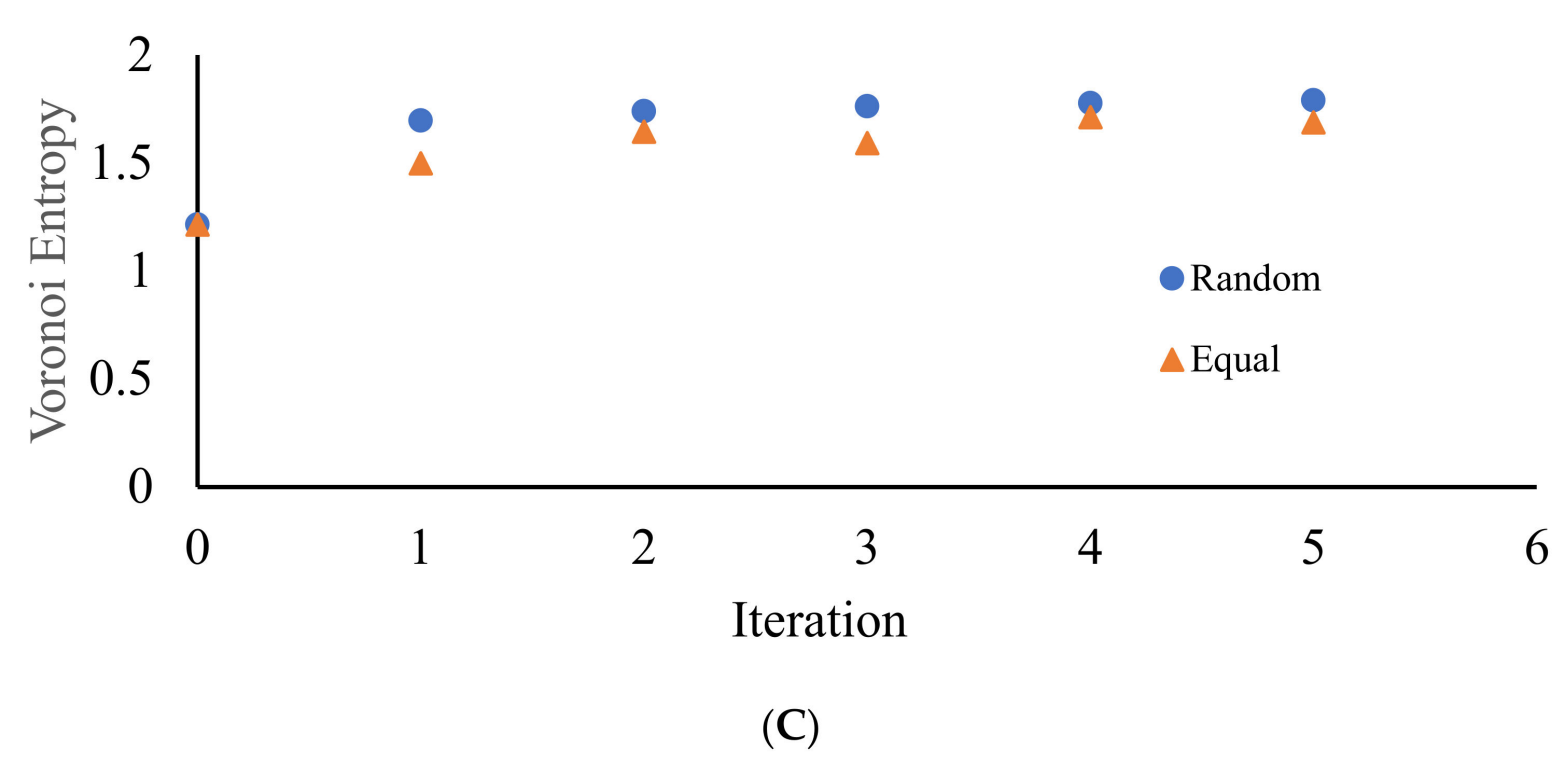
VE calculated for the patterns emerging from a random initial distribution of the points. The number of repetitions is shown on the *X*-axis. (**A**) VE for the seeds arising from the equal partition of the edges for 10 original sets of random points. (**B**) VE for the seeds arising from the random partition of the edges for 10 original sets of random points. (**C**) Comparison of VE for one of the sets (set 25Rand_06). Blue circles depict VE for the seeds arising from the random partition of the edges; orange triangles correspond to the VE established for the Voronoi diagrams emerging from the equal partition of the edges into two segments.

Quantitative characterization of the reported Voronoi diagrams was performed with the calculation of the Voronoi entropy (VE) and continuous measure of symmetry (CSM) of the diagrams. The Shannon (Voronoi) entropy of the given set of points located on a plane is defined as

(2) $$S_{vor}=-\sum_{i}P_{i}lnP_{i}$$

where $P_{i}$ is the fraction of polygons possessing *n* edges for a given Voronoi diagram (also called the coordination number of the polygon) and i is the total number of polygon types with a different number of edges. The summation in Equation (1) is performed from *i* = 3 to the largest coordination number of any available polygon, e.g., to *i* = 6, if a polygon with the largest number of edges is a hexagon. For the details of calculation of CSM, introduced into Section 2.3, see reference [29]. Let us start from the binary dividing of the initial Voronoi diagrams, which generated the patterns in Figure 3 and Figure 4. The results of the calculations are illustrated in Figure 7.

It is clearly recognized from Figure 7 that regardless of the VE of the original set, with the increasing number of repetitions (iterations), VE approaching the close asymptotic values, namely the random partition of the edges, yielded the asymptotic value of the VE $S_{vor,\;random}^{asymp}≅1.8$, whereas the equal partition of the edges yielded $S_{vor,\;2}^{asymp}≅1.7.$ This means that an increase in the number of iterations yields more disordered patterns (as estimated with the informational (Shannon) measure of the distribution of the Voronoi polygons) approaching a fixed asymptotic value of the VE. It is noteworthy that VE of the aforementioned patterns is close to that corresponding to completely random patterns [37], namely $S_{vor,random}≅1.71$. Thus, we necessarily conclude that VE does not distinguish positional ordering, namely the spherulite-like ordering, inherent for the patterns depicted in Figure 3 and Figure 4.

**Figure 8:**
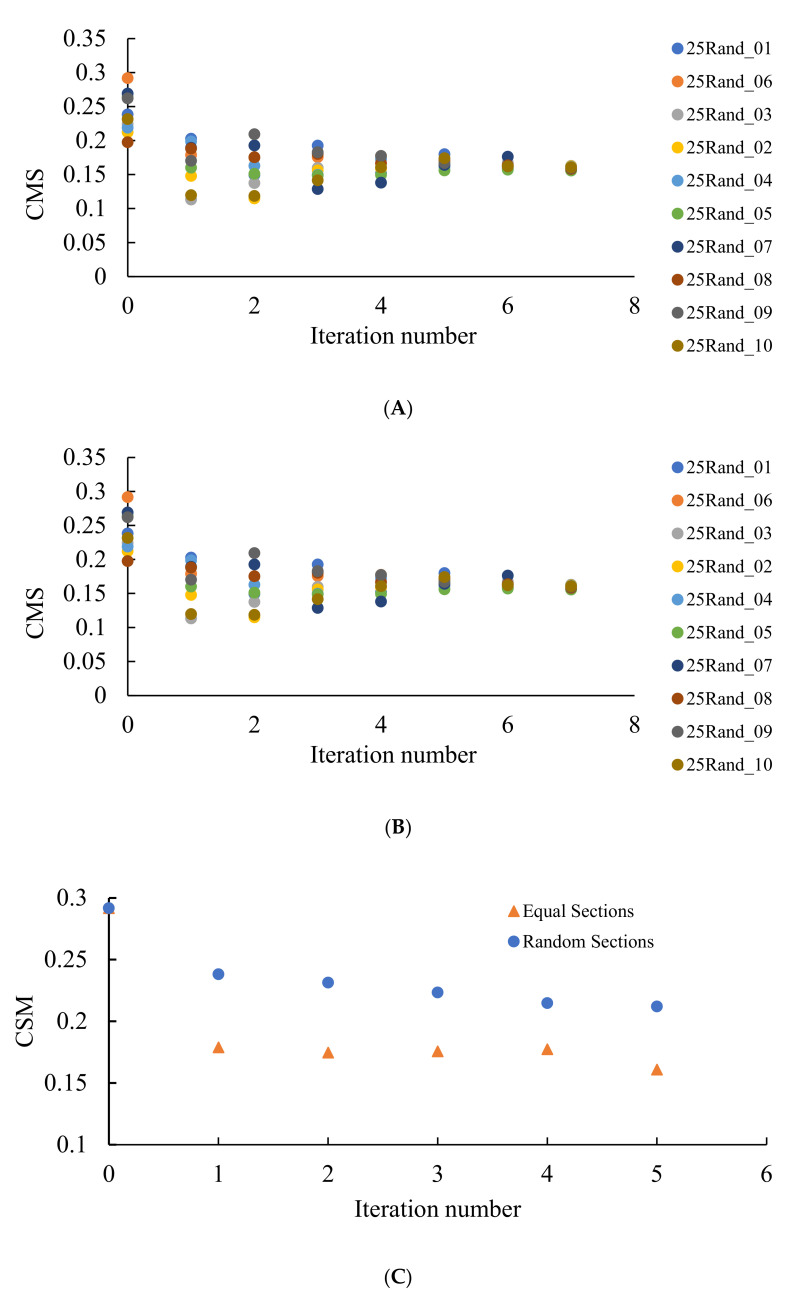
Continuous measure of symmetry (CSM) calculated for the patterns emerging from the random initial distribution of the points. The number of repetitions is shown on the *X*-axis. (**A**) CSM for 10 original sets of seeds after division into two equal parts is depicted. (**B**) CSM for 10 original sets of seeds after division into two random parts is shown. (**C**) Comparison of the CSM in the same original set of seeds after division of edges into two equal or random parts. Blue circles depict CSM for the seeds arising from the random partition of the edges; orange triangles correspond to seeds emerging from the equal partition of the edges into two segments.

The continuous measure of symmetry of the same patterns demonstrated the opposite asymptotic behavior, i.e., CSM decreased with the number of repetitions (iterations) of the suggested process, as it is recognized from Figure 8. In other words, polygons in the patterns become more symmetric with the number of iterations. We conclude that VE and CSM demonstrated the opposite tendency when calculated for the same patterns, demonstrating spherulite-like short ordering, when edges of the Voronoi tessellations were divided into two segments.

**Figure 9:**
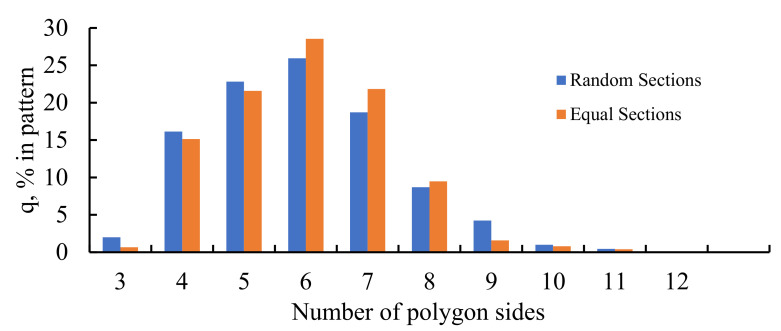
Histogram representing the distribution of polygons in the Voronoi diagrams emerging from the seeds dividing the edges of the preceding Voronoi tessellation into two parts: blue columns represent the random partition; orange columns represent the equal partition of the edges.

It was also instructive to study the distribution of polygons within the studied Voronoi tessellations. The histograms representing these distributions are supplied in Figure 9. It is seen that the most popular polygon in these Voronoi tessellation is a hexagon; this situation is typical for random patterns. This result arises from the general topological properties of random 2D patterns as discussed in reference [11].

Now, consider the properties of the diagrams established for the patterns obtained when the edges of the pristine Voronoi tessellations were divided into three equal segments, or alternatively the same edges were randomly divided into three segments.

**Figure 10:**
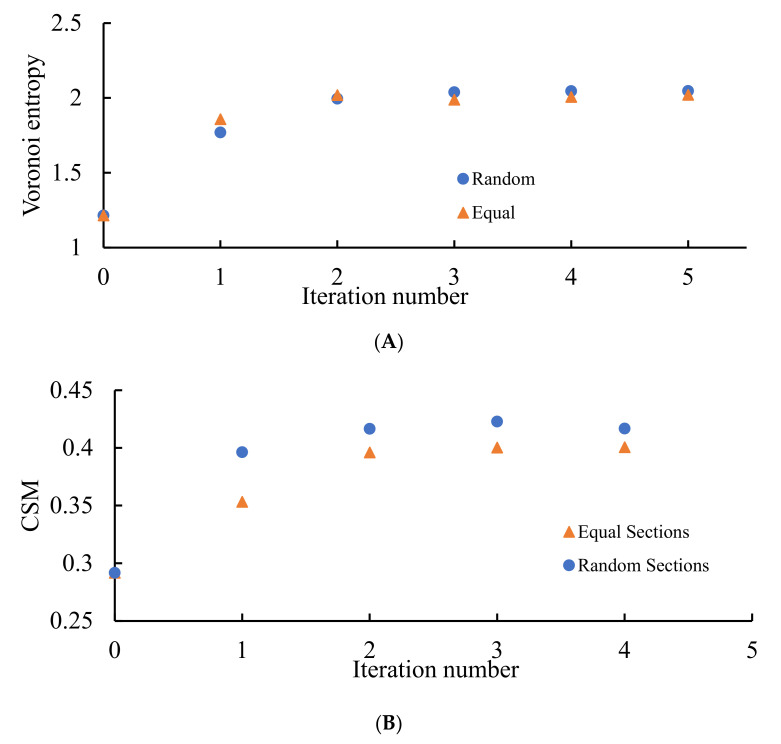
VE and CSM calculated for the patterns emerging from the random initial distribution of the points. (**A**) Blue circles depict VE for the seeds arising from the random partition of the edges into three segments; orange triangles correspond to the VE established for the Voronoi diagrams emerging from the equal partition of the edges into three segments. (**B**) Blue circles depict CSM for the seeds arising from the random partition of the edges into three segments; orange triangles correspond to CSM established for the Voronoi diagrams emerging from the equal partition of the edges into three segments. The number of repetitions is shown on the *X*-axis.

It should be emphasized that in this case, the Voronoi entropy is growing with the number of iterations of the discussed process under attaining the asymptotic value of $S_{vor}=2.03\pm 0.02$, as shown in Figure 10A; CSM demonstrated the same asymptotic behavior, attaining with the number of iterations the value of CSM≅0.4, as shown in Figure 10B. This is true for both random and non-random partitions of the edges of the pristine Voronoi tessellations. This means that the asymptotic behavior of patterns, shown in Figure 5 and Figure 6, demonstrates ordering recognized by both VE and CSM. Consider that CSM in this case is twice larger than that established for the patterns emerging from dividing the edges of the Voronoi diagrams into two parts as shown in Figure 8, pointing to the less symmetrical nature of the polygons constituting the patterns. This quantitative result evidences the elongated shapes of the lamellae, inherent for the patterns corresponding to the partition of the edges into three parts, when compared to the same shapes corresponding for the patterns arising from the partition of the edges into two parts. This means that the sides of lamellae are more different in their length.

**Figure 11:**
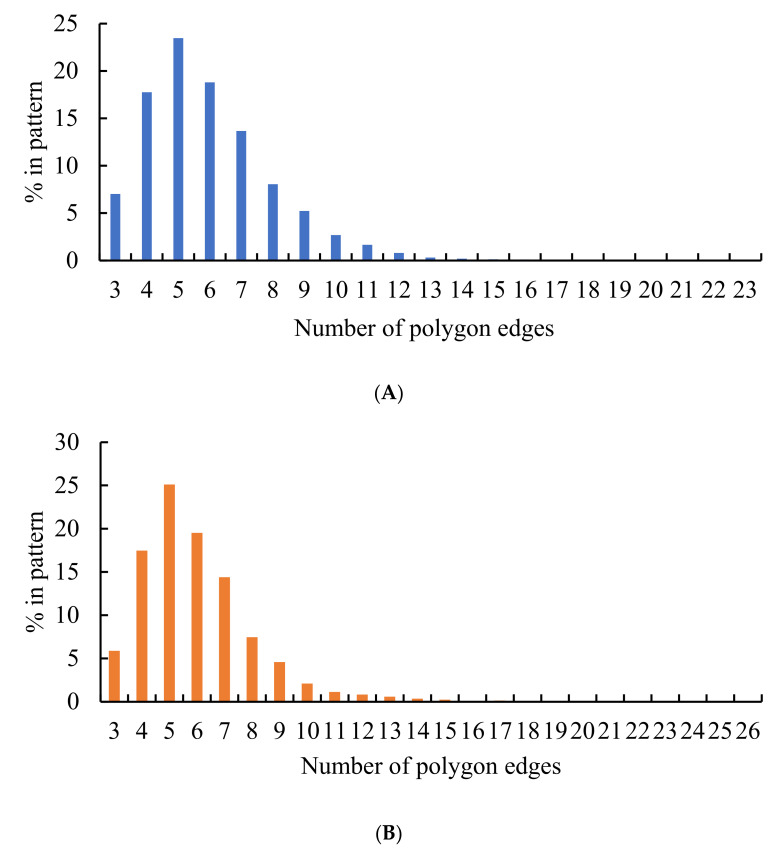
Histograms representing the distribution of polygons in the Voronoi diagrams emerging from dividing the edges of the preceding Voronoi tessellation into three parts: (**A**) Blue columns represent the random partition; (**B**) Orange columns represent the equal partition of the edges.

Now, consider the distribution of polygons in the patterns arising from dividing the edges of the Voronoi diagrams into three segments, illustrated in Figure 11. The similarity of the distributions corresponding to the random and non-random partitions of the edges catches the eye. One of the surprising features of these distributions calls for explanations: the most common polygons in both of these distributions are pentagons; it should be stressed the that the most popular polygon in random 2D patterns is a hexagon [11]. This observation also evidences the ordered nature of the patterns shown in Figure 5 and Figure 6. The presence of exotic polygons possessing a large number of edges in the patterns arising from the partition of edges of the pristine Voronoi tessellation into three segments, recognized from Figure 11B, is also noteworthy.

### 3.3. Physical Interpretation of the Self Organization

Possible interpretation of the observed self-organization may be suggested within the physical model suggested in reference [11]. Let us view the initial Voronoi diagram, such as that depicted in Figure 1 as a random two-dimensional soap foam. The total “surface energy” *U* of such a soap is defined by the total length of all cell edges.

(3) $$U=2\gamma \sum_{edges}l_{i}$$

where γ denotes the “surface tension” (the dimensions of γ are $\left[\gamma \right]=\frac{J}{m}$, consider that the pattern is a two-dimensional one), and $l_{i}$ is the length of cell edge *i*. When the edges forming the Voronoi polygons are divided into two or three parts, equally or randomly, $\sum_{edges}l_{i}=const$ takes place. This implies U=const; thus, the formation of the next generation of the Voronoi diagrams takes place under the constant surface energy of the foam, which is not kept constant within the entire iterative process. Thus, the entire iterative process, suggested in the manuscript, may be interpreted as follows: we start from the random 2D Voronoi foam, generated by the randomly distributed nuclei. In the next step, new nuclei precipitate on the edges of the polygons, forming the foam. The process of precipitation keeps the “total surface energy” of the foam constant. Physically, precipitation of new nuclei (seeds) on the edges of polygons sounds natural and thermodynamically justified. The system rearranges itself around new nuclei. Thus, self-ordered polymer-like lamellae appear in the pattern. In the realm of polymer crystallization, the introduced process corresponds to the “secondary nucleation” scenario introduced by Lauritzen and Hoffman in their pioneering paper on the theory of polymer crystallization from dilute solution, published in 1960 [38]. Secondary is the nucleation which occurs on the *surfaces* of crystals of the material [38,39]. Thus, new seeds appearing on the edges of the Voronoi tessellation are seen as the centers of the secondary crystallization [39]. Secondary crystallization in polymer results in formation of lamellae constituting polymer spherulites, as shown in references [38,39]. The consequent iterations of the introduced procedure of generation of Voronoi diagrams correspond to the stages of secondary, tertiary, etc., crystallization occurring in the polymer melt or solution. It is noteworthy that lamellar structures were registered not only in polymers, but also in the metallic glasses [40,41]. The physical reasoning of such short-range ordering calls for additional insights; however, it is reasonable to suggest that they emerge from the secondary crystallization process.

## 4. Conclusions

We investigated the properties of patterns arising from the Voronoi tessellations stemming from the random initial distribution of points on the plane [11,12,13,14,15,16,17]. The following mathematical procedure was applied: at the first stage, the Voronoi tessellation generated by a random set of seeds was built. At the next stage, the following iterative procedure was applied to the initial random Voronoi tessellation: the edges forming the Voronoi polygons were divided into two or three parts, equally or randomly. The dividing points were then taken as the new seeds for the new Voronoi tessellations (the former seeds were excluded). This new diagram, in turn, was subjected to the division of the edges of the polygons. The mathematical experiment was repeated for 10 different initial sets of random points. The results of the experiment were quite surprising: equal or random dividing of the edges of the Voronoi polygons gave rise to ordered patterns, strongly resembling those registered for linear semi-crystalline polymers such as polyethylene or polypropylene and metallic glasses [30,31,32,34,35]. Lamellae and spherulite-like structures, perceived as the evidence of short-range ordering, systematically, appeared in the eventual patterns. Thus, clearly distinguished 2D ordering emerged from the aforementioned mathematical procedure applied to the initially random set of points. We quantified the ordering with Voronoi entropy and the continuous measure of symmetry [24,25,26,27,28,29]. The Shannon (Voronoi) entropy and the continuous measure of symmetry of the addressed patterns demonstrated pronounced asymptotic behavior, while approaching the close asymptotic values with the increase in the number of iteration steps. Voronoi entropy grew with the number of repetitions (iterations), whereas the continuous measure of symmetry of the same patterns demonstrated the opposite asymptotic behavior, i.e., it decreased with the number of repetitions (iterations) of the introduced process. In other words, polygons in the patterns become less ordered from the point of view of the Voronoi entropy, but more symmetric (as quantified with the continuous measure of symmetry) with the number of iterations. We necessarily conclude that the notion of ordering is not well defined. The Shannon (Voronoi) entropy, which increased in our mathematical experiments, is not sensitive to the lamellae and spherulite-like ordering, clearly recognized in our model experiments. So, the Voronoi entropy is not an unambiguous measure of order in the 2D patterns. The more symmetrical patterns may demonstrate relatively higher values of the Shannon (Voronoi) entropy. These conclusions support the findings established under the analysis of the Penrose tiling and ordering registered for the levitating water clusters [28,29]. It is reasonable to suggest that the introduced mathematical iterative procedure possesses substantial common features with the secondary crystallization process, giving rise to the formation of spherulites in the polymer melts and solutions [38,39].

## Data Availability

Not applicable.
